# A Two-Layer Structural Key Framework for Linking Compound Identifiers and MS/MS Evidence in Spectral Database Curation

**DOI:** 10.3390/metabo16070435

**Published:** 2026-06-23

**Authors:** Kaiwen Deng, Ran Liu, Ruiping He, Li Chen

**Affiliations:** Shanghai Key Laboratory of Metabolic Remodeling and Health, Institute of Metabolism & Integrative Biology, Fudan University, Shanghai 200433, China

**Keywords:** metabolomics, MS/MS spectral databases, InChIKey, stereochemistry, molecular connectivity

## Abstract

**Background**: MS/MS spectral databases provide reference spectra for compound identification in metabolomics studies. Their utility depends on clear links among compound identifiers, chemical structures, and MS/MS evidence, yet these links are often complicated by database-specific identifiers, heterogeneous structural representations, and stereochemical specifications. **Methods**: Here, we present a two-layer structural key framework for linking compound identifiers and MS/MS evidence through standardized structures. Reported SMILES were standardized and converted into InChIKey-derived stereo keys and connectivity keys using a Python-based RDKit workflow. **Results**: As illustrated using stereoisomeric cases such as L- and D-proline, the stereo key layer preserves compound identifiers and metadata at the stereo level, whereas the connectivity key layer groups comparable MS/MS evidence at the molecular connectivity level. In a database-scale application, 217,920 HMDB compound entries were organized into 216,783 stereo keys and 196,512 connectivity keys, and 144,591 spectra from the spectrum-centered MoNA database were incorporated into the HMDB-centered framework, increasing MS/MS evidence coverage, particularly at the molecular connectivity level. **Conclusions**: Together, this framework links compound identifiers, standardized structures, and MS/MS evidence at the stereo and connectivity levels, providing a bidirectionally traceable system for spectral database curation without forcing connectivity-level MS/MS evidence into stereo-specific compound identities.

## 1. Introduction

MS/MS spectral databases are central reference resources for compound annotation in mass-spectrometry-based metabolomics. These annotated compounds then need to be linked to compound databases that provide chemical and biological metadata for downstream interpretation [1,2,3]. Community and curated resources, including HMDB [4], MoNA [5], GNPS [6], ReSpect [7], and others, have substantially expanded access to compound-associated MS/MS evidence. However, the utility of these resources depends not only on the number of spectra they contain but also on whether spectra can be clearly linked to compound identifiers in compound databases [1,8].

A first challenge is that compounds may be represented by database-specific identifiers, common names, synonyms, submitted SMILES strings, or systematic identifiers, and these descriptors can be ambiguous or inconsistent within and across small-molecule databases [9,10,11]. In addition, alternative salt forms, counterions, protonation states, tautomeric forms, and differences in structure drawing or SMILES encoding can fragment records that refer to the same core chemical structure into seemingly separate compounds, dispersing biological annotations across entries that should be interpreted together [12,13,14]. Chemical structure standardization pipelines, together with structure-derived identifiers such as InChI and InChIKey, provide practical routes for harmonizing heterogeneous structural representations and enabling links across resources [15,16,17,18].

A second challenge is that compound identity and MS/MS evidence do not always operate at the same level of structural resolution. Stereoisomers can represent distinct compounds with different biological properties, but conventional MS/MS fragmentation often provides limited stereochemical discrimination [19,20,21]. When MS/MS evidence that primarily supports molecular connectivity is directly linked to a stereospecific compound record, a practical consequence of this resolution mismatch is the risk of false stereoiso-mer-level annotation.

To address these challenges, we developed a two-layer structural key framework for linking compound identifiers and MS/MS evidence through standardized structures. In this framework, reported SMILES strings are standardized into core chemical structures and then converted into InChIKey-derived structural keys. The stereo key layer retains compound identifiers, names, structures, and associated metadata at the stereo level, whereas the connectivity key layer groups comparable MS/MS evidence at the molecular connectivity level. This design separates compound-level identity tracking from MS/MS evidence organization while preserving traceable links among compound identifiers, standardized structures, and individual MS/MS records. We first illustrate the need for this two-layer organization using representative examples of heterogeneous structural representations and stereoisomeric compounds with overlapping MS/MS evidence. We then apply the framework to the HMDB compound database and MoNA spectral database to show how standardized structural keys can bridge these resources without enforcing a one-to-one relationship between compound entries and MS/MS records.

## 2. Materials and Methods

### 2.1. Data Sources

The Human Metabolome Database (HMDB; version: 5.0, distributed date: 2 November 2021) was used for the construction of the standardized structure database, where 217,920 metabolite structures and metadata were parsed in total.

Public MS/MS spectra were obtained from the HMDB MS/MS database and the MassBank of North America (MoNA) LC-MS/MS spectral exports. HMDB MS/MS spectra were parsed from the 2023 HMDB MS/MS release, yielding 65,029 source spectra, while MoNA spectra were parsed from positive- and negative-ion LC-MS/MS MSP export snapshots dated 25 July 2024, yielding 144,591 source spectra.

### 2.2. Structure Standardization

The SMILES field was used as input for structural standardization. Using Python (version: 3.12.12), recorded SMILES were standardized with RDKit (version: 2025.03.4) by SMILES parsing, largest fragment selection, functional group normalization, charge neutralization with Uncharger, and stereo-retaining tautomer canonicalization. Canonical isomeric SMILES, InChIKey, molecular formulas, exact mass and formal charge were generated from the standardized molecule, with RDKit default sanitization, aromaticity and canonicalization settings. Isotopic labels were retained throughout the process. Neutral formulas and neutral mass were derived from the standardized formulas and formal charge. Records were flagged when RDKit failed to generate a standardized SMILES from reported SMILES strings (Table S1).

### 2.3. InChIKey-Based Compound Grouping

For InChIKey-based grouping, the stereo key was defined as the first two blocks of the standardized InChIKey, and the connectivity key was defined as the first 14-character block.

Standardized HMDB records were grouped by stereo key and connectivity key respectively, and the first HMDB accession in each group was used as the representative structure entry for records sharing the same layer of keys. Original HMDB accessions and compound metadata were preserved in a linked metadata index, allowing source records to remain traceable after being grouped into representative structure entries.

### 2.4. Experimental Spectra Acquisition

The 293T cell lysate was used to obtain experimental MS/MS spectra from a real biological sample. Data-dependent MS/MS acquisition was used in both positive and negative electrospray ionization modes. Data were acquired on a Thermo Scientific Orbitrap Exploris 480 mass spectrometer (Thermo Fisher Scientific, Waltham, MA, USA) and converted to centroided open-format files. The inspected acquisition records used full-scan MS over *m*/*z* 70 to 1050, followed by HCD MS/MS with a recorded collision energy value of 40.0 and an isolation width of approximately 1.7 *m*/*z*.

Experimental MS/MS scans were linked to chromatographic features by precursor *m*/*z* and retention time agreement. For experimental spectra used in the amino acid example, MS/MS scans were assigned when the precursor *m*/*z* differed by no more than 0.015 Da and the scan retention time fell within the feature window.

### 2.5. MS/MS Spectral Similarity Comparison

Spectral similarity between experimental and reference spectra and among reference spectra was calculated using a modified cosine score from Matchms [22]. Fragment intensities were transformed by square root before scoring.

For two spectra *A* and *B*, the score was calculated as follows:

$$S(A,B)\;=\;\frac{\sum_{(i,j)\in M}\;w_{i}w_{j}}{\sqrt{\sum_{i}\;{w_{i}}^{2}}\sqrt{\sum_{j}\;{w_{j}}^{2}}},\;w_{i}\;=\;{I_{i}}^{0.5}$$

Here, *M* denotes the selected set of non-overlapping matched peak pairs. Fragment peaks were allowed to match either directly within 0.02 Da or after shifting by the precursor *m*/*z* difference between the two spectra. Candidate peak matches were ranked by the product of transformed intensities, and non-overlapping peak pairs were selected greedily. Spectra with empty peak lists or zero fragment-intensity norms were assigned a score of zero.

### 2.6. Two-Layer Assignment of MS/MS Spectra to Standardized Structures

Public MS/MS spectra from HMDB and MoNA were assigned to representative HMDB structure entry through mapping via stereo key and connectivity key respectively. Both keys were generated from structure descriptors embedded in the spectral metadata using the same structure standardization procedure applied to HMDB records described above. The resulting stereo key and connectivity key were compared with the HMDB-derived structure index at separate levels. A matching stereo key was used for stereo-aware assignment to a standardized HMDB structure entry, whereas a connectivity key was matched for connectivity-level MS/MS assignment.

### 2.7. Parsing MS/MS Spectral Records from HMDB and MoNA

All HMDB and MoNA spectral records were used as input for the curation workflow. Each spectrum then passed through three ordered stages, namely structure mapping, quality control filtering, and deduplication. In the first stage, spectra without a matching HMDB structure at the connectivity level were excluded.

In the second stage, the mapped spectra passed through a series of quality control filters, where spectra were removed when the recorded ion mode was neither positive nor negative, when the fragment peak count exceeded 1000, when the precursor *m*/*z* or adduct annotation was missing, or when the adduct was incompatible with the recorded ion mode. Spectra were further removed when the observed precursor *m*/*z* differed by at least 0.5 Da from the value calculated for the assigned standardized structure and recorded adduct. The 0.5 Da window was used because precursor *m*/*z* values may contain limited decimal precision. Theoretical precursor *m*/*z* values were recalculated from standardized structures and recorded adduct information.

Deduplication was further performed on spectra passing quality control. Spectral records with identical ion mode, fragment peak count, and connectivity-level assigned structure were grouped and deduplicated. Within each group, MS/MS fragment peaks were compared pairwise, and spectra were treated as duplicates when all fragment *m*/*z* differences were no greater than 0.0005 and intensity differences were no greater than 0.05. One representative spectrum was retained from each duplicated group. Detailed stepwise accounting of removals throughout the database cleaning and curation workflow is provided in Supplementary Table S3.

## 3. Results

### 3.1. Heterogeneous Structural Representations Can Be Harmonized Through Core Structure Standardization

Compounds are commonly described by database-specific compound identifiers, reported names, and submitted SMILES strings. However, these fields do not necessarily correspond one-to-one to distinct core chemical structures. In biomedical research, downstream interpretation often relies on the core structure of a compound, but alternative salt forms, protonation states, or tautomeric representations may fragment one compound into multiple database records.

To examine this issue, we summarized representative classes of heterogeneity in structural representation and applied a standardization workflow to convert reported SMILES into standardized core SMILES (Table 1). This workflow first resolved purely notational differences by mapping alternative SMILES reported for benzoic acid to the same standardized core structure. It then reconciled differences arising from chemical form. For example, salt form entries such as citric acid and monosodium citrate were harmonized after removal of the sodium counterion. Protonation state variants, represented by pyridoxamine and pyridoxaminium, and keto-enol tautomeric variants, represented by phenylpyruvic acid and enolphenylpyruvate, were likewise standardized to shared core representations. In contrast, stereochemical specifications were retained because they can define distinct compound identities and remain important for compound-level annotation and metadata tracking.

We therefore applied a core structure standardization workflow to harmonize heterogeneous compound entries while preserving stereochemical information for compound-level annotation and metadata tracking.

### 3.2. Stereoisomeric Entries Can Provide Highly Similar MS/MS Evidence

Although stereochemical specification is important to define compound identity, conventional MS/MS fragmentation primarily reflects bond connectivity and fragment composition and therefore may not provide sufficient evidence to distinguish stereoisomeric compounds.

**Figure 1:**
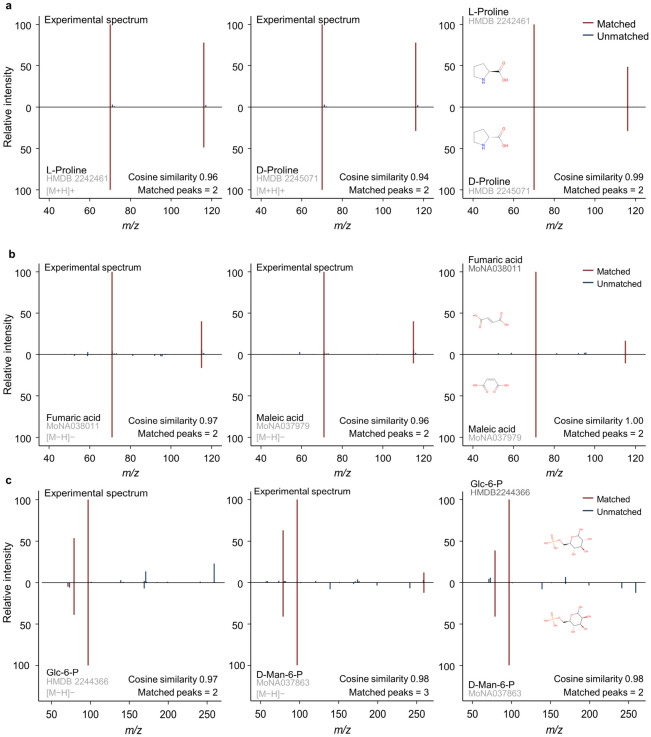
Mirror plot comparison of MS/MS spectra from stereoisomeric compound pairs. Within each panel, the left and middle plots compare the experimental spectrum (top) with each reference spectrum (bottom), and the right plot compares the two reference spectra directly, with 2D structures identifying the isomers. (**a**) Proline enantiomers in positive ion mode. Experimental proline [M+H]^+^ spectrum compared with HMDB L-proline and D-proline reference spectra, followed by the direct L-proline versus D-proline comparison. (**b**) The geometric isomers fumaric acid (trans) and maleic acid (cis) in negative ion mode ([M−H]^−^). (**c**) The C-2 epimers glucose 6-phosphate (Glc-6-P) and D-mannose 6-phosphate (D-Man-6-P) in negative ion mode ([M−H]^−^). Paired spectra are displayed above and below the baseline. Red and blue peaks indicate matched and unmatched fragment ions, respectively; cosine similarity, a measure of spectral resemblance from 0 to 1, is shown in each plot. Gray text gives the source database accession for each reference spectrum.

We examined this issue using proline as a representative example, and cosine similarity was used to quantify the similarity between two MS/MS spectra. An experimental positive-mode [M+H]^+^ proline MS/MS spectrum showed high similarity to database reference spectra annotated as both L-proline and D-proline (Figure 1a). In parallel, direct comparison between the L-proline and D-proline reference spectra showed highly similar fragmentation patterns, with shared major product ions and comparable relative intensity distributions (Figure 1a). Thus, while L-proline and D-proline represent distinct stereo-specific compound entries, their MS/MS spectra provide largely overlapping evidence under the examined acquisition conditions.

Beyond the enantiomeric proline case, we further inspected other diastereomeric cases, including maleic acid/fumaric acid representing a cis/trans pair (Figure 1b) and glucose-6-phosphate/Mannose-6-phosphate representing a multi-stereocenter pair (Figure 1c). These additional cases also showed high cosine similarity scores, indicating that this issue also exists in diastereomeric cases.

Together, the highly similar MS/MS evidence between stereoisomers supports grouping MS/MS spectra from stereoisomeric entries at the connectivity level.

### 3.3. A Two-Layer Structural Key Framework Links Stereo-Specific Compound Information and Connectivity-Level MS/MS Evidence

We next implemented a two-layer structure-based framework to organize compound entries and their associated MS/MS spectra. Reported SMILES strings were first converted into standardized core SMILES and then into InChIKey representations. The stereo key layer was used to retain stereochemical information and compound-level metadata, whereas the connectivity key layer was used to group spectral entries with the same molecular connectivity.

**Figure 2:**
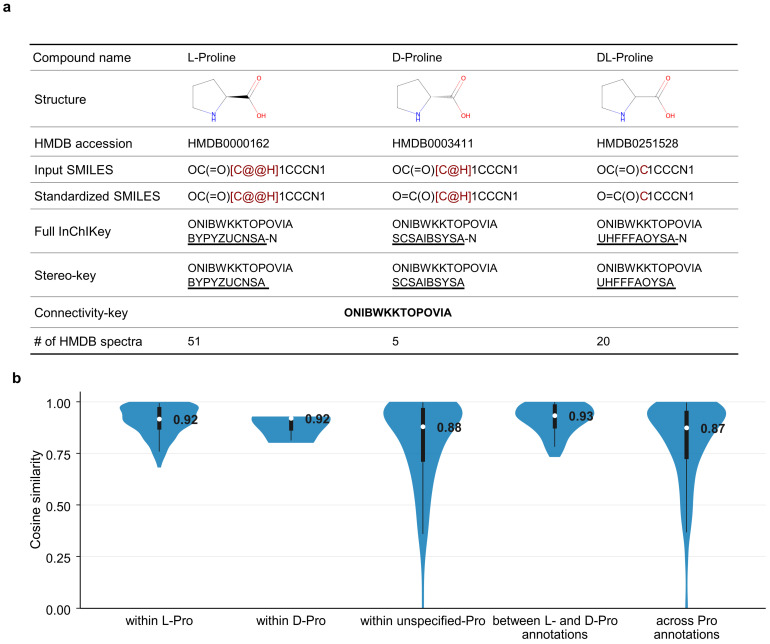
Two-layer InChIKey organization of proline metadata and MS/MS evidence. (**a**) Proline-related HMDB records are assigned to stereo key and connectivity key layers after standardized SMILES processing. Red SMILES characters and underlined InChIKey blocks indicate differences in stereochemical notation. (**b**) Pairwise cosine similarity score for positive-ion [M+H]+ HMDB spectra mapped to the proline connectivity key. Violin plots show score distributions across comparison groups, namely within L-Pro (n = 171), within D-Pro (n = 3), within unspecified-Pro (n = 190), between L- and D-Pro annotations (n = 57), and across Pro annotations (n = 861), where n is the number of pairwise comparisons; black bars indicate interquartile ranges; white points and numeric labels indicate medians; the lower and upper whiskers indicate the 5th–95th percentiles.

Using proline-related entries as an example, L-proline, D-proline, and DL-proline were assigned distinct stereo keys but shared the same connectivity key (Figure 2a). This organization preserved their stereo-specific compound records while placing their associated MS/MS spectra under a shared connectivity key group. We then evaluated MS/MS similarity across all L-proline, D-proline, and DL-proline spectra from HMDB in [M+H]^+^, including within-form and between-form comparisons. These spectra showed generally high similarity across these comparison groups, further supporting the need to group MS/MS spectra at the connectivity key level (Figure 2b).

Together, this two-layer framework organizes compound information and MS/MS evidence at complementary structural levels. Stereo key is retained for compound annotation and metadata tracking, while connectivity key groups MS/MS spectra at the molecular connectivity level.

### 3.4. The Two-Layer Framework Links HMDB Compound Records with MoNA Spectrum Records Through Standardized Structures

HMDB is organized around compound entries with identifiers, structures, biomedical metadata, and MS/MS spectra, whereas MoNA is organized around individual MS/MS spectra with compound annotations. We next explored whether the two-layer framework, built on standardized core structures, could link these two databases.

**Figure 3:**
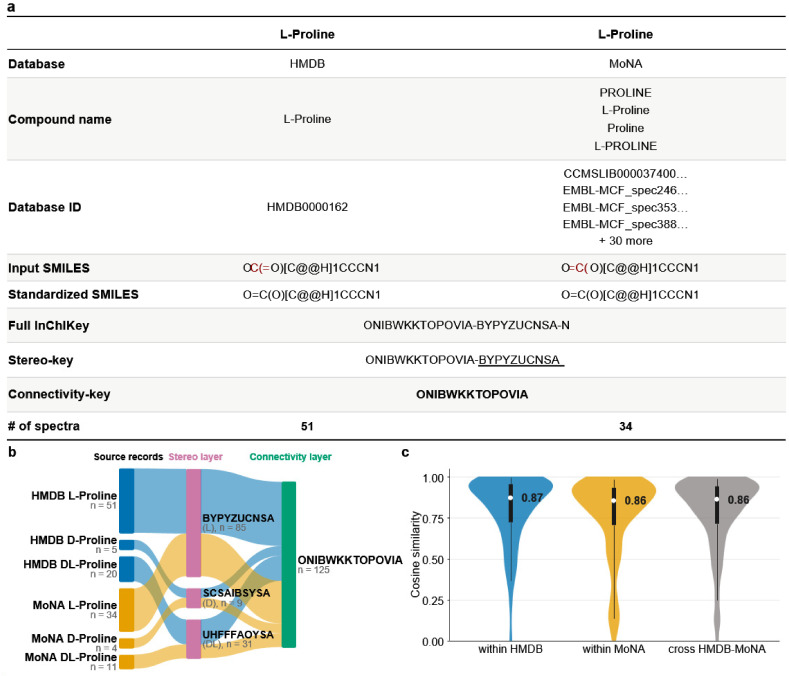
Cross-database spectrum linking through proline’s structural keys. (**a**) Source record mapping for L-proline based on standardized SMILES, InChIKey, stereo key, and connectivity key. Red SMILES characters and underlined InChIKey blocks indicate differences in stereochemical notation. (**b**) Sankey diagram showing the assignment of L-, D-, and DL-proline annotations to stereo key and connectivity key groups; flow width represents the number of spectra. (**c**) Pairwise cosine similarity score distributions for spectra mapped to the proline connectivity key, grouped as spectra within HMDB (n = 861), spectra within MoNA (n = 703), and cross-database comparisons (n = 1596), where n is the number of pairwise comparisons. Violin plots show score distributions; bars indicate interquartile ranges; points indicate medians; the lower and upper whiskers indicate the 5th–95th percentiles.

For each MoNA spectrum record, the associated structural annotation was first converted into standardized SMILES and then into stereo key and connectivity key representations. Using proline as an example, the HMDB L-proline compound entry and MoNA L-proline spectrum records were assigned to the same standardized SMILES, full InChIKey, stereo key, and connectivity key (Figure 3a). D-proline and DL-proline mappings showed similar results (Table S2). This two-layer framework organized 51, 5, and 20 HMDB spectra and 34, 4, and 11 MoNA spectra of L-proline, D-proline, and DL-proline, respectively, into three stereo key groups that shared the same proline connectivity key (Figure 3b).

We further compared the MS/MS spectra within HMDB, within MoNA, and between HMDB and MoNA entries under this connectivity key (Figure 3c). This subset included 42 HMDB spectra and 38 MoNA spectra, yielding 861 within-HMDB, 703 within-MoNA, and 1596 cross-source pairwise comparisons. The corresponding median cosine scores were 0.873, 0.858, and 0.862, respectively, supporting the use of the connectivity key as a common structure layer for organizing comparable proline MS/MS evidence across databases.

This example demonstrates that shared structural keys can connect MoNA spectrum records to HMDB compound records without forcing a one-to-one database match. Stereo keys support links to stereo-specific compound annotations, whereas the connectivity key provides a common structural layer for organizing comparable MS/MS spectra across sources.

### 3.5. Database-Scale Implementation Links MoNA MS/MS Evidence to HMDB Compound Records

After demonstrating with proline-related records that MoNA spectral entries can be linked to HMDB compound identifiers through shared structural keys, we applied the same framework at the database scale. Instead of constructing a single compound identifier and MS/MS table, we organized database information into two linked curation outputs. The first was a compound identifier-structure table, which maps HMDB compound identifiers, names, structures, and metadata to stereo keys and connectivity keys. The second was an MS/MS spectrum-structure table, which maps individual HMDB and MoNA spectra to the same structural keys. Because both tables retained the same stereo keys and connectivity keys, compound identifiers and MS/MS spectra could be traced bidirectionally while being organized at the structural level most appropriate for each type of information.

**Figure 4:**
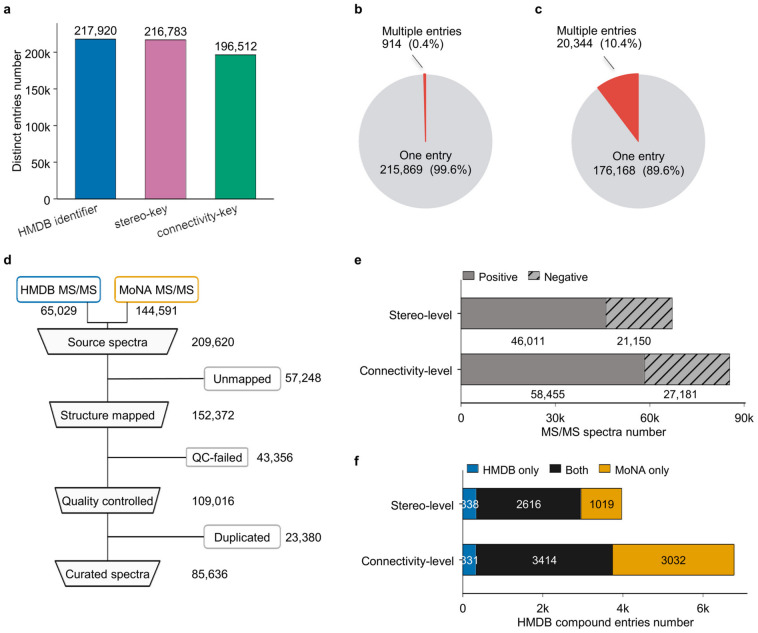
Two-layer organization of MS/MS evidence across full HMDB and MoNA. (**a**) Distinct HMDB compound identifiers, stereo keys, and connectivity keys in the HMDB compound structure table. (**b**) Distribution of the number of HMDB compound entries associated with each stereo key. (**c**) Distribution of the number of HMDB compound entries associated with each connectivity key. (**d**) Spectrum curation workflow for HMDB and MoNA MS/MS records, including structure assignment, quality control, removal of duplicate records, and generation of the final indexed spectrum set. (**e**) Indexed MS/MS spectra grouped by ionization mode and summarized at the stereo key and connectivity key levels. (**f**) HMDB compound entries with linked MS/MS evidence grouped by evidence source and summarized at the stereo key and connectivity key levels. Source categories indicate compound entries supported by HMDB spectra only, MoNA spectra only, or spectra from both databases.

We first established the HMDB-centered compound identifier-structure table from HMDB compound entries. Reported SMILES strings were pre-cleaned and converted into standardized core SMILES, followed by assignment of stereo keys and connectivity keys. This workflow organized 217,920 HMDB compound identifiers into 216,783 distinct stereo keys and 196,512 distinct connectivity keys while retaining their original compound identifiers and names (Figure 4a). At the stereo key level, 215,869 stereo keys corresponded to a single HMDB compound identifier, whereas 914 stereo keys grouped multiple identifiers, reflecting condensation of heterogeneous structural representations with retained stereochemistry (Figure 4b). Meanwhile, 20,344 connectivity keys grouped multiple HMDB compound identifiers, compared with 176,168 one-entry connectivity keys, reflecting the consolidation of compounds that shared the same core connectivity but differed in stereochemical specification (Figure 4c).

We next generated the MS/MS spectrum-structure table by incorporating spectra from both HMDB and MoNA. The source collection contained 65,029 HMDB spectra and 144,591 MoNA spectra. After basic structure mapping, quality control, and duplicate removal, 85,636 curated structure-linked spectra remained (Figure 4d, Table S3). Across the structure-linked set, 67,161 spectra retained stereo key assignments, while 18,475 additional spectra were incorporated at the connectivity key level only (Figure 4e). Retained HMDB spectra were linked to both stereo keys and connectivity keys through their associated HMDB compound entries. For MoNA spectra, some entries were assigned to both stereo keys and connectivity keys, whereas others were incorporated at the connectivity key level when stereo-specific information was incomplete or unavailable. This allowed MoNA spectra to be organized within the HMDB-centered structural framework without forcing all spectrum records into stereo-specific compound identities.

Finally, we examined how MoNA spectra contributed to the MS/MS coverage of HMDB-linked structures after being incorporated into the same structural key system. At the stereo key level, 3973 HMDB compound entries (~1.8%) had linked MS/MS evidence, including 2616 entries with both HMDB and MoNA evidence and 1019 entries supported only by MoNA spectra. At the connectivity key level, 6777 HMDB compound entries (~3.1%) had linked MS/MS evidence, including 3414 entries with both sources and 3032 entries supported only by MoNA spectra. Despite the percentage coverage being limited, this comparison showed that incorporating MoNA increased the number of HMDB-linked structures associated with MS/MS evidence, with a more pronounced contribution at the connectivity key level (Figure 4f).

Together, this database-scale implementation demonstrates how the two-layer framework manages compound-centered and spectrum-centered database information without enforcing a one-to-one relationship between compound identifiers and MS/MS records. By using structural keys as the organizing units, the workflow enables HMDB compound identifiers and HMDB/MoNA MS/MS spectra to remain bidirectionally traceable while allowing HMDB-linked MS/MS coverage to be expanded through incorporation of MoNA spectra.

## 4. Discussion

This study addresses an important problem in MS/MS spectral database curation: compound identifiers, structural representations, and MS/MS evidence do not always align at the same level of structural resolution. Existing resources have greatly expanded the availability of reference spectra and associated compound information [3,4,6,23], yet more spectra do not by themselves ensure that compound identities and spectral evidence are linked at the most appropriate structural level. The main contribution of our framework is to traceably link compound database records and MS/MS spectral records through two-layer standardized structural keys, without forcing connectivity-level MS/MS evidence into stereo-specific compound identity. Recent database curation efforts have improved the reliability and usability of spectral resources through data quality improvement, metadata repair, public spectrum harmonization, redundancy removal, and reproducible database cleaning [22,24,25]. Our framework is complementary to these efforts by adding two-layer structural key fields to database curation outputs, so that spectrum-side records can be linked to compound-side identifiers through both stereo key and connectivity key layers. Compared with workflows that use connectivity information for spectral database deduplication [26] or connectivity structure-centric MS/MS evidence searching [12], our explicit bidirectional mapping allows connectivity-level MS/MS evidence to traceably link to all associated stereo-specific compound entries. Specifically, established structural representations and structure-derived identifiers, including SMILES and InChIKey, are used to generate two linked curation outputs: a compound identifier-structure table and an MS/MS spectrum-structure table. In this design, a compound record is first mapped to a stereo key and a connectivity key, allowing MS/MS evidence linked to the same connectivity key to be retrieved from spectral databases. Conversely, when an experimental MS/MS spectrum matches a reference spectrum, the corresponding connectivity key can retrieve all associated stereo keys and compound records rather than directly overclaiming a stereo-specific assignment. Thus, the two-layer structural keys provide bidirectional traceability between compound database and spectral database while making explicit the structural resolution at which each type of database information should be interpreted.

The broader value of this framework is that it provides a scalable linking strategy rather than a fixed spectral resource. Because SMILES and InChIKey are widely supported across chemical and metabolomics databases, they provide a shared structural language for cross-resource harmonization [15,27]. As demonstrated by the HMDB-MoNA integration, structural keys can bridge compound databases and MS/MS spectral databases with different record architectures, extending the number of HMDB entries that have MS/MS evidence coverage. Considering the MS/MS evidence coverage is still limited relative to known compounds, it is therefore anticipated that the same integration strategy can be applied beyond the specific resources used in this study, including other public resources, institutional databases, project-specific compound lists, or internally acquired spectral databases for a more comprehensive spectral database curation [12].

Several boundaries of the framework should also be noted. First, connectivity-level grouping should be interpreted as an evidence organization layer, not as mandatory merging of stereoisomeric identities or as a rule for downstream annotation. When stereoisomers can be distinguished by MS/MS or by orthogonal evidence, such as chromatographic separation, chiral methods, derivatization, ion mobility, or specialized MS/MS strategies, the stereo key layer still preserves the ability to link evidence to more specific compound identities [19,20,28,29]. Second, the framework itself does not alter the quality of reported structures and metadata. Incorrect SMILES, incomplete stereochemical specifications, inconsistent adduct annotations, or insufficient acquisition metadata may lead to incorrect structural key assignments and affect downstream curation. Therefore, this framework should be used as a traceable structural organization layer that is complementary to, rather than a replacement for, existing spectral curation tools and annotation workflows.

## 5. Conclusions

This study presents a two-layer structural key framework for linking compound identifiers, standardized structures, and MS/MS evidence in spectral database curation. By separating stereo-key-based compound metadata from connectivity-key-based MS/MS evidence, the framework helps prevent overassignment of stereo-specific metabolite identities when the supporting MS/MS evidence primarily reflects molecular connectivity. Importantly, stereochemical information is retained and remains traceable, as each connectivity key is linked to its associated stereo keys and compound records, allowing users to retrieve either a specific stereochemically defined entry or the complete set of stereochemical alternatives sharing the same molecular connectivity, depending on the annotation context. Applied to HMDB and MoNA, this framework enabled bidirectional tracing between compound-centered and spectrum-centered resources and expanded HMDB-linked MS/MS evidence coverage, particularly at the connectivity key level. Overall, the approach provides a scalable organization strategy for the curation of metabolomics spectral databases.

## Figures and Tables

**Table 1:** Heterogeneity of structural representation resolved by core structure standardization.

Representation Class	Compound Name	Identifier	Reported SMILES	Standardized Core SMILES
Alternative SMILES writing convention	Benzoic acid Benzoic acid	PubChemCID 243 HMDB0001870	C1=CC=C(C=C1)C(=O)O OC(=O)C1=CC=CC=C1	O=C(O)c1ccccc1
Salt forms	Citric acid Monosodium citrate	HMDB0000094 HMDB0303750	O=C(O)CC(O)(CC(=O)O)C(=O)O [Na+]. OC(=O)CC(O)(CC(O)=O)C(O)=O	O=C(O)CC(O)(CC(=O)O)C(=O)O
Protonation state	Pyridoxamine Pyridoxaminium	HMDB0001431 HMDB0062696	CC1=C(O)C(CN)=C(CO)C=N1 CC1=C(O)C(C[NH3+])=C(CO)C=N1	Cc1ncc(CO)c(CN)c1O
Keto-enol tautomer	Phenylpyruvic acid Enolphenylpyruvate	HMDB0000205 HMDB0012225	OC(=O)C(=O)CC1=CC=CC=C1 OC(=O)C(\O)=C\C1=CC=CC=C1	O=C(O)C(=O)Cc1ccccc1
Stereochemistry	L-Aspartic acid D-Aspartic acid	HMDB0000191 HMDB0006483	N[C@@H](CC(O)=O)C(O)=O N[C@H](CC(O)=O)C(O)=O	N[C@@H](CC(=O)O)C(=O)O N[C@H](CC(=O)O)C(=O)O

## Data Availability

The reference spectra data used in this study were obtained from public databases, including HMDB and MoNA. Example code and dataset are available via Zenodo at https://zenodo.org/records/20603181 (accessed on 9 June 2026). The deposited materials include code and scripts, processed stereo key and connectivity key tables, spectrum-structure mapping outputs, example proline-related HMDB and MoNA records, and README files. Other data supporting the findings of this study are provided in the article and Supplementary Materials.

## Supplementary Materials

The following supporting information can be downloaded at: https://www.mdpi.com/article/10.3390/metabo16070435/s1, Table S1: HMDB structural records for which RDKit could not generate a standardized structure, with the reason for failure and the handling applied; Table S2: Source record mapping for D-proline and DL-proline in the proline connectivity key group; Table S3: Removed spectra and their corresponding reasons during library curation.
