# Supplementary material for: A Two-Layer Structural Key Framework for Linking Compound Identifiers and MS/MS Evidence in Spectral Database Curation
Source: Metabolites. 2026 Jun 23;16(7):435. doi: 10.3390/metabo16070435 (PMC13414330; doi:10.3390/metabo16070435)
Supplement: Supplementary file 1 [file metabolites-16-00435-s001.zip › metabolites-4362262-supplementary.pdf]

A two-layer structural-key framework for linking compound identifiers and MS/MS evidence in spectral database curation

Supplementary Materials

This supplementary file contains the supplementary tables associated with the manuscript, including Table S1, Table S2 and Table S3.

Supplementary Tables

Table S1. HMDB structural records for which RDKit could not generate a standardized structure, with the reason for failure and the handling applied.

**Outcome of HMDB structure standardization**	**HMDB structural records (n)**	**% of total records (n = 217,920)**
Standardized successfully, connectivity-key assigned	217,879	99.98%
Source record contained no structure string	24	0.011%
Source SMILES could not be parsed by RDKit	17	0.008%
**Total processed**	**217,920**	**100.00%**

Table S2. Source-record mapping for D-proline and DL-proline in the proline connectivity-key group.

	**D-Proline**	**D-Proline**	**DL-Proline**	**DL-Proline**
Database	HMDB	MoNA	HMDB	MoNA
Compound name	D-Proline	PROLINE	DL-Proline	Proline<br>PROLINE<br>L-PROLINE<br>proline
Database ID	HMDB0003411	MoNA033048<br>MoNA033055<br>MoNA038244<br>MoNA038785	HMDB0251528	CCMSLIB00000577898<br>CCMSLIB00000578125<br>CE000622<br>CE000633<br>CE000636<br>CE000639<br>ML005301<br>MT000067<br>MoNA002226<br>MoNA002227<br>MoNA002228
Input SMILES	OC(=O)[C@H]1CCCN1	O=C(O)[C@H]1CCCN1	OC(=O)C1CCCN1	O=C(O)C1CCCN1
Standardized SMILES	O=C(O)[C@H]1CCCN1	O=C(O)[C@H]1CCCN1	O=C(O)C1CCCN1	O=C(O)C1CCCN1
Full InChIKey	ONIBWKKTOPOVIA-SCSAIBSYSA-N		ONIBWKKTOPOVIA-UHFFFAOYSA-N	
Stereo-key	ONIBWKKTOPOVIA-SCSAIBSYSA		ONIBWKKTOPOVIA-UHFFFAOYSA	
Connectivity-key	ONIBWKKTOPOVIA			
# of spectra	5	4	20	11

Table S3. Removed spectra and their corresponding reasons during library curation.

Curation step	Removal reason	Removed spectra (HMDB)	Removed spectra (MoNA)	Total spectra removed per step, n (% of all source spectra)
Unmapped	Missing SMILES or other structural metadata	120	240	57,248 (27.31%)
	RDKit failed to generate a connectivity-key	16	16	
	Connectivity-key absent in HMDB structures	0	56,856	
QC-failed	Ion mode is neither positive nor negative	199	0	43,356 (20.67%)
	More than 1,000 fragment peaks in one spectrum	39	3,816	
	Missing adduct	30,535	3,472	
	Recorded adduct contradicts with ion mode	2,206	940	
	Missing precursor m/z	0	108	
	Recorded precursor m/z inconsistent with theoretical value by at least 0.5 Da	358	1,683	
Duplicated	Identical spectra deduplicated*	1,260	22,120	23,380 (11.15%)
Total		34,733	89,251	123,984 (59.13%)

* When duplicate spectra from HMDB and MoNA were present in the same deduplication group, HMDB spectra were retained preferentially, which contributed to the larger number of MoNA spectra removed at this step.
